# Genome-Wide Identification and Functional Characterization of the *Dof* Gene Family in Flax (*Linum usitatissimum*)

**DOI:** 10.3390/ijms27094126

**Published:** 2026-05-05

**Authors:** Chenmeng Xu, Limin Wang, Zhao Dang, Wenjuan Li, Wei Zhao, Yaping Xie, Yan Wang, Jianping Zhang, Yanni Qi

**Affiliations:** Institute of Crop, Gansu Academy of Agricultural Sciences, Lanzhou 730070, China; 18735906104@163.com (C.X.); liminwang@aliyun.com (L.W.); dangzhao@gsagr.cn (Z.D.); liwenjuan@gsagr.ac.cn (W.L.); zhaowei@gsagr.ac.cn (W.Z.); xieyp2012@126.com (Y.X.); 1073323120598@st.gsau.edu.cn (Y.W.)

**Keywords:** flax, Dof transcription factor, *LuDof* genes, phylogenetic analysis, abiotic stress responses, expression analysis

## Abstract

DNA-binding with one finger (Dof) transcription factors are plant-specific regulators of growth, development, and stress responses. Despite extensive characterization in various species, *Dof* genes in flax (*Linum usitatissimum* L.), an important oil and fiber crop, remain largely uncharacterized. Through genome-wide identification and comprehensive characterization of the *Dof* gene family in flax, this study identified 47 *LuDof* genes in the high-oil Longya-10 variety, distributed non-uniformly across 15 chromosomes. Phylogenetic analysis grouped these genes into 12 distinct clusters, reflecting evolutionary conservation and lineage-specific characteristics, including the absence of *LuDof* members in Group XII. Gene structure and conserved motif analyses revealed strong structural conservation, particularly within the canonical zf-Dof domain. Segmental duplication was identified as the primary driver of *LuDof* family expansion, with all paralogous pairs evolving under strong purifying selection. Collinearity analysis revealed that 80.9% of *LuDof* genes have homologs in other species, highlighting strong evolutionary conservation. Promoter analysis identified multiple hormone- and stress-responsive elements, and qRT-PCR under drought, heat, cold, and methyl jasmonate (MeJA) treatments confirmed their roles in environmental stress responses. Transcriptome profiling indicated their involvement in stem and capsule development. This study represents the first systematic characterization of the evolution, structure, and functional potential of the flax *Dof* gene family, establishing a foundation for functional studies and for developing genetically superior, stress-tolerant flax varieties.

## 1. Introduction

*Linum usitatissimum* L., commonly referred to as flax and a member of the genus *Linum*, is a crop of global significance owing to its dual utility as a source of high-quality edible oil and natural fiber. Thought to have originated in the Near East, flax is now extensively cultivated worldwide, with major producers including Canada, the United States, India, Russia, Germany, Northern Europe, and China [1,2,3].

A substantial body of research has established that flaxseeds are abundant in essential nutrients, including dietary fiber, lignans, vitamins, and polyunsaturated fatty acids [4]. In recent years, growing global emphasis on health promotion and preventive nutrition has intensified interest in the nutritional properties and associated health benefits of flaxseed. In addition to its nutritional significance, flax fiber is regarded as one of the oldest and most highly valued natural fibers, owing to its exceptional tensile strength, comfort, environmental sustainability, and versatility across a wide range of applications [5]. These attributes render flax highly suitable for a wide range of applications in textiles, composite materials, and various industrial products. In light of the growing demand for flax-based products across the food, health, and industrial sectors, there is a pressing demand to improve its agronomic performance, particularly with respect to yield, stress tolerance, and quality of its oil and fiber. Therefore, elucidation of the genetic mechanisms underlying these agronomic traits is essential for the genetic improvement of flax through advanced breeding techniques and biotechnological interventions. The DNA-binding with one zinc finger (*Dof*) gene family represents a group of plant-specific transcription factors (TFs) that play critical roles in the regulation of stress-responsive signaling pathways and key metabolic processes, including lipid and cellulose biosynthesis [6]. Therefore, the identification and characterization of the *Dof* gene family in flax may provide critical insights into the molecular mechanisms underlying these agronomic traits, thereby enabling the identification of potential targets for the genetic improvement of flax.

Dof TFs constitute a distinct subfamily of zinc finger proteins, typically comprising 200–400 amino acid residues [7]. These proteins are characterized by the presence of a conserved, cysteine-rich single zinc finger domain, referred to as the Dof domain, which mediates both DNA binding and protein–protein interactions [8]. Structurally, Dof proteins consist of two primary functional regions, including a highly conserved N-terminal DNA-binding domain that contains the Dof domain (~52 amino acids). This domain adopts a C2C2-type zinc finger configuration characterized by the CX2CX21CX2C motif [9] and specifically recognizes core DNA sequences such as 5′-AAAG-3′ and 5′-CTTT-3′ [10]. Four cysteine residues within this zinc finger structure coordinate a single Zn2+ ion, which is essential for maintaining the structural integrity and functional activity of Dof TFs [11]. The Dof domain exhibits dual functionality, binding core DNA sequences to regulate downstream gene expression while also mediating interactions with other proteins to modulate plant growth, development, and stress responses [12]. In addition, Dof TFs possess a variable C-terminal transcriptional activation region, which exhibits low sequence conservation among Dof family members and interacts with multiple regulatory proteins, including MYB, WRKY, and bZIP TFs [8], thereby contributing to the functional diversity of Dof proteins. The structural variability of this domain enables differential protein–protein interactions, thereby enabling Dof TFs to participate in a wide range of signaling and regulatory networks [13].

Dof TFs have emerged as key regulators of diverse physiological and cellular processes in plants, including phytohormone signaling, response to abiotic stress, seed germination, floral transition, photosynthetic regulation, and light-mediated development [7,8,11,14]. Following the initial discovery of the first *Dof* gene in maize (*Zea mays*) in 1995 [15], subsequent studies have identified and characterized *Dof* gene families across a wide range of plant species [7]. Systematic characterization of all 36 Dof TFs in *Arabidopsis thaliana* revealed that CDF3 exhibits dual functionality, enhancing tolerance to abiotic stresses such as drought, cold, and osmotic stress while concurrently delaying flowering time [8,9]. The *Dof* gene family in rice (*Oryza sativa*) comprises 30 members, including *OsDof15*, which regulates the elongation of primary roots through modulation of ethylene-mediated meristematic cell proliferation [9,16]. Previous studies have identified 24 *Dof* genes in tomato (*Solanum lycopersicum*), among which *TDDF1* promotes early flowering and enhances tolerance to drought and salinity stress [17,18]. The *ZmDof22* gene in maize has been shown to enhance drought tolerance by modulating stomatal aperture and increasing the activities of antioxidant enzymes [19], whereas *ZmDof36* promotes the biosynthesis of grain starch while concurrently suppressing the accumulation of soluble and reducing sugars [20]. Similarly, overexpression of the carrot *RsDof33* gene in *Arabidopsis* led to reduced rosette leaves, delayed flowering, and increased anthocyanin content. It also upregulated the expression of genes involved in auxin synthesis (AtYUC2), auxin transport (AtPIN4), leaf shape development (AtKNAT2), and the anthocyanin synthesis pathway (AtPAL, AtCHS, AtDFR, etc.) [21]. The evolutionary conservation and functional diversification of Dof TFs are further substantiated by their characterization in a wide range of economically important species, including cereals such as maize (*Zea mays*) [22] and barley (*Hordeum vulgare*) [23]; horticultural crops such as kiwi (*Actinidia* sp.) [24] and watermelon (*Citrullus lanatus*) [25]; medicinal plants such as ginseng (*Panax ginseng*) [26]; root crops such as cassava (*Manihot esculenta*) [10], and leafy vegetables such as spinach (*Spinacia oleracea*) [27]. These comprehensive studies collectively demonstrate that *Dof* genes constitute a conserved yet functionally versatile gene family that has evolutionarily diversified to regulate fundamental physiological processes across the plant kingdom.

Recent advancements in high-throughput sequencing technologies and comparative genomics have enabled the systematic identification and comprehensive characterization of *Dof* gene families across diverse plant species. Despite extensive studies in model plants and major crops, the *Dof* gene family in flax remains largely uncharacterized, highlighting a significant gap in our understanding of transcriptional regulation in this economically important fiber and oilseed crop.

In this study, an integrated bioinformatics approach was employed to identify *Dof* genes in flax at the genome-wide level, followed by comprehensive analyses of their phylogenetic relationships, gene structures, conserved protein domains, and expression patterns across various tissues and under different stress conditions. This study represents the first systematic and comprehensive characterization of the *Dof* gene family in flax, providing evolutionary insights through comparative phylogenetic analyses and generating expression profile data suggestive of their potential roles in stress responses. The findings presented in this study provide a robust foundation for future functional investigations of Dof-mediated regulatory networks in flax, particularly concerning their potential roles in abiotic stress responses and other agronomically important traits. These insights may further inform the development of targeted genetic improvement strategies aimed at enhancing the cultivation of flax under adverse environmental conditions.

## 2. Results

### 2.1. Identification of Dof Genes in Flax

Flax is divided into oil flax and fiber flax. Longya-10 is an oil flax variety, while Heiya-14 is a fiber flax variety. A total of 47, 48, and 45 *Dof* genes were identified in the Longya-10, Heiya-14, and pale flax (*Linum bienne*) varieties, respectively, through BLASTp (https://blast.ncbi.nlm.nih.gov/Blast.cgi (accessed on 18 December 2025)) and conserved domain analysis. The 47 *Dof* genes identified in the Longya-10 cultivar were sequentially designated *LuDof1*–*LuDof47* based on their chromosomal locations (Table 1). Comparative physicochemical characterization of Dof TFs across the three flax genomes revealed broadly consistent parameter ranges. Specifically, the Dof TFs in the Longya-10, Heiya-14, and pale flax varieties spanned 171–470, 171–491, and 171–471 amino acids, respectively, with corresponding molecular weights (MW) of 18,483.96–51,215.01 Da, 18,498.03–53,808.21 Da, and 18,483.96–51,197.91 Da, respectively, and isoelectric points (pI) of 4.45–9.75, 4.51–9.49, and 4.45–9.85, respectively. Additionally, the grand average of hydropathicity (GRAVY) values for all the identified Dof proteins were negative (<0), indicating their hydrophilic nature. Further analysis of subcellular localization conducted using online websites indicates that all Dof proteins in flax are localized within the cell nucleus (Table 1).

### 2.2. LuDof Genes Sequence Analysis

**Figure 1:**
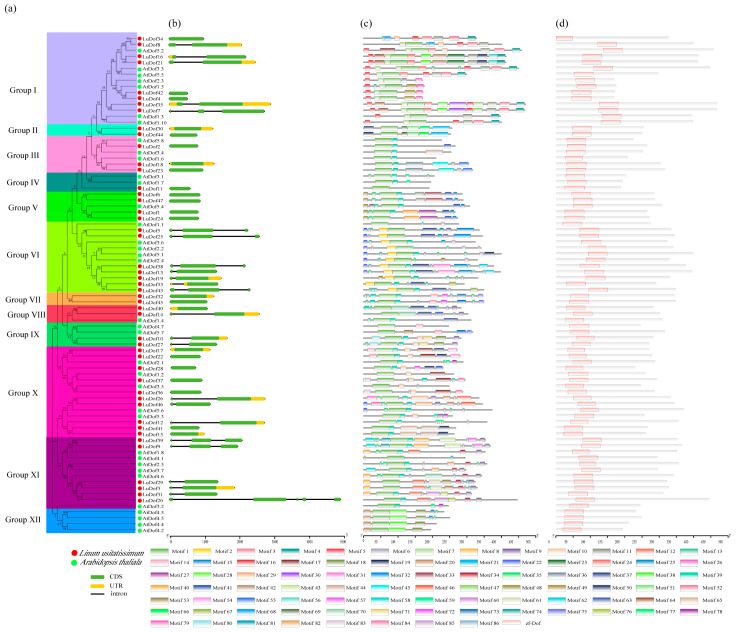
Unrooted phylogenetic tree, conserved motifs and domain analysis of Dof family. (**a**) Phylogenetic tree of LuDof proteins. (**b**) Gene structure of *LuDof* genes. (**c**) Motif composition of LuDof proteins identified using MEME. (**d**) Analysis of the domain structure of the *LuDof* gene. Different colors represent distinct motifs.

The *Dof* gene family in flax was further characterized through phylogenetic analysis using the Dof protein sequences from flax and *Arabidopsis* sp., which resolved into 12 distinct clusters (Figure 1a). Among these, Groups I and X contained the highest number of Dof proteins, with 15 members each, whereas Groups II and VII were the smallest, each comprising only two Dof proteins exclusively derived from flax. Group I included eight and seven Dof proteins from flax and *Arabidopsis* sp., respectively, whereas Group X comprised ten and five Dof proteins from flax and *Arabidopsis* sp., respectively. Notably, Group XII lacked any Dof proteins from flax, suggesting that this clade is likely absent in the flax genome.

Gene structure analysis revealed that *LuDof20* exhibited the most complex architecture among the 47 *LuDof* genes, containing five exons and four introns, and representing both the highest exon count and longest genomic span observed. Among the 47 *LuDof* genes, 24 were intronless (single-exon), 15 contained two exons, and 7 contained three exons (Figure 1b). Comparative analysis of *Dof* gene structures in pale flax and Heiya-14 revealed variations in exon–intron organization, with pale flax comprising 17 single-exon, 21 two-exon, five three-exon, and two four-exon genes, whereas Heiya-14 comprised 22 single-exon, 19 two-exon, six three-exon, and one five-exon gene (Tables S1 and S2). Motif analysis further identified 48 distinct motifs, each exclusively present in two members of the *LuDof* family.

Comparative analyses of the conserved motifs in flax and *Arabidopsis* sp. identified a total of 86 distinct conserved protein motifs (Tables S4). The minimum length of the motif is 6, and the E-value used during the search process is “inf” (which stands for “infinity”). Among these 86 motifs, one core motif (Motif 1) was identified as a Dof domain containing a C_2_C_2_ zinc finger structure, representing a conserved DNA-binding region of the Dof transcription factor family. Additionally, several functional motifs were discovered, including a glutamine-rich transcriptional activation region (Motifs 2 and 13), arginine/lysine-rich nuclear localization signals (Motifs 5, 26, and 35), an acidic amino acid-rich protein interaction region (Motif 7), a polyhistidine metal-binding region (Motif 6), a glycine-rich flexible linker region (Motif 9), and a potential amphipathic α-helix (Motif 8). Collectively, these motifs reveal the molecular mechanism of Dof proteins as transcription factors, encompassing nuclear localization, DNA binding, transcriptional regulation, and potential phosphorylation modification. Notably, Motif 1 was present in all Dof proteins analyzed in this study, with the exception of LuDof34. Among these, 48 motifs were each exclusively detected in two members of the LuDof family, corresponding to paralogous gene pairs. Additionally, several motifs exhibited group-specific distribution patterns across the Dof subfamilies. For instance, Motifs 4, 5, 7, 16, 24, 26, 28, and 64 were exclusively detected in Group I, whereas Motif 48 was restricted to Group V. Motif 85 was detected only in Group VI and was exclusively present in the *Arabidopsis AtDof* family, whereas Motif 38 was specific to Group XII. Among the LuDof proteins, four members, namely, LuDof7, LuDof16, LuDof21, and LuDof35, contained the highest number of motifs (12 each), whereas LuDof11 harbored the fewest, with only a single conserved motif.

To gain deeper insights into the structural features of the Dof domain, a comparative sequence analysis of LuDof and AtDof proteins was performed. All the analyzed proteins were found to contain the canonical zf-Dof domain, demonstrating that this DNA-binding domain is highly conserved across the Dof protein families of flax and *Arabidopsis* sp. (Figure 1d).

### 2.3. Chromosomal Distribution of LuDof Genes and Collinearity Analysis

**Figure 2:**
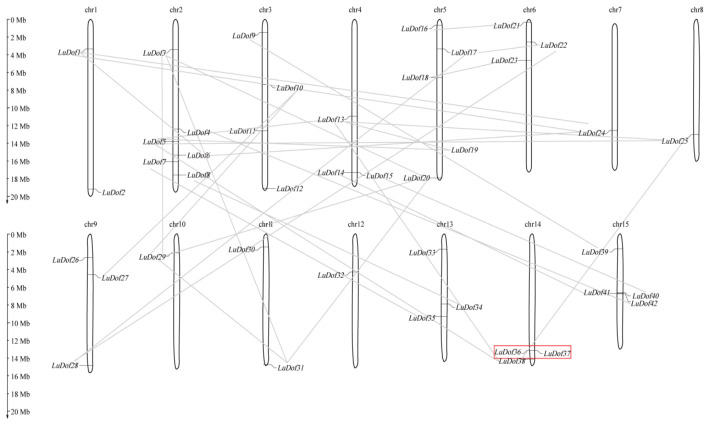
Chromosome location of *LuDof* genes. Red rectangle and gray lines show the gene pairs underwent TD and SD, respectively.

Analysis of the chromosomal distribution of *LuDof* genes revealed that 42 genes were non-uniformly distributed across 15 chromosomes (Figure 2), whereas the remaining five genes (*LuDof43*–*LuDof47*) could not be mapped to any specific chromosome. Chromosome 2 harbored the highest number of *LuDof* genes (*n* = 6), whereas chromosomes 7, 8, 10, and 12 each harbored only a single *LuDof* gene, and the remaining chromosomes carried two to four *LuDof* genes. Gene duplication analysis identified 39 duplicated gene pairs, comprising 38 segmental duplication (SD) events involving 42 *LuDof* genes, which collectively 89.4% of all duplicated genes, and a single tandem duplication (TD) event that gave rise to the *LuDof36/37* gene pair. These results suggest that the expansion of the *LuDof* gene family is predominantly driven by SD events (Table 2). We further estimated the nonsynonymous (Ka) and synonymous (Ks) substitution rates for each duplicated gene pair, and the resulting Ka/Ks ratios were used to infer the selective pressures underlying their evolutionary divergence. The Ka/Ks ratios for all the duplicated gene pairs were consistently < 1, ranging from 0.035 to 0.638, indicating that these *LuDof* paralogs have been subjected to strong purifying selection throughout their evolutionary history. We additionally identified nine duplicated gene pairs (*LuDof3/LuDof31*, *LuDof5/LuDof13*, *LuDof5/LuDof19*, *LuDof5/LuDof25*, *LuDof6/LuDof47*, *LuDof18/LuDof23*, *LuDof19/LuDof43*, *LuDof24/LuDof47*, and *LuDof33/LuDof43*) involving a total of 14 *LuDof* genes, with Ks values ranging from 0.625 to 0.863. These values suggest that these duplication events likely coincided with an early whole-genome duplication (WGD) event (Ks = 0.77) [28]. The corresponding divergence times were estimated to be approximately 51–71 million years ago (Mya), whereas the remaining gene pairs exhibited divergence times ranging from 23 to 273 Mya.

**Figure 3:**
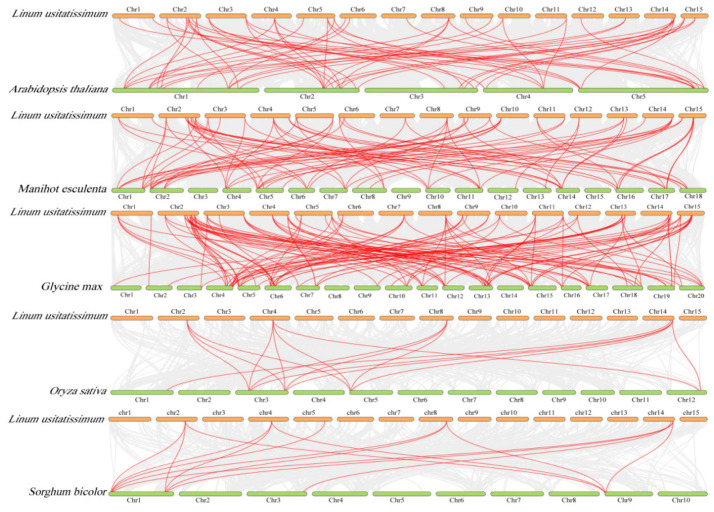
Collinearity analysis of *Dof* genes between flax and five other plant species (*Arabidopsis*, soybean, cassava, rice and sorghum). Gray lines indicate syntenic blocks between the flax genome and those of other species, whereas red lines represent collinear *Dof* gene pairs.

To further elucidate the evolutionary mechanisms shaping the *LuDof* gene family, a comparative collinearity analysis was conducted between flax and five additional eudicot and monocot species, including *Arabidopsis* sp., cassava, soybean, rice, and sorghum (Figure 3 and Table S5). The findings revealed that homologs of 38 (80.9%) *LuDof* genes were identified in at least two of the analyzed species, with each homolog forming one to five collinear orthologous gene pairs. Notably, conserved syntenic orthologs of five *LuDof* genes, namely, *LuDof5*, *LuDof13*, *LuDof25*, *LuDof36*, and *LuDof38*, were identified in all the species analyzed herein, Among these, *LuDof5*, *LuDof13*, and *LuDof25* were consistently iconserved in *Arabidopsis* sp., cassava, and sorghum. Moreover, each *LuDof* gene was associated with up to five homologous counterparts across the five eudicot and monocot species compared in this study. Synteny analysis revealed that flax exhibited the strongest collinear relationships with the eudicot species, with 56–120 collinear orthologous gene pairs, whereas the weakest relationships were observed with the monocot species, with only 14–15 pairs identified.

### 2.4. Phylogenetic Analysis of the Dof Family

**Figure 4:**
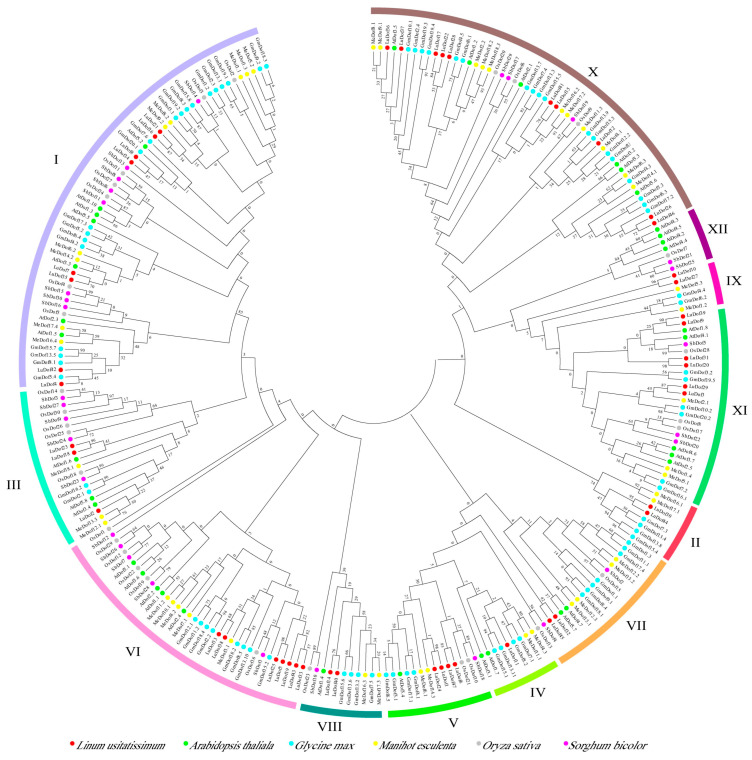
Phylogenetic tree of Dof proteins from flax, *Arabidopsis*, soybean, cassava, rice and sorghum. Dofs were divided into 12 subgroups, and each subgroup was identified by a different color. Dof proteins obtained from different species were marked by circles filled with different colors.

To investigate the evolutionary conservation of the *Dof* gene family during the domestication of flax, a phylogenetic analysis was performed using Dof protein sequences from the cultivated varieties Longya-10 and Heiya-14, together with those from their wild-type progenitor, pale flax (*Linum bienne*) (Supplementary Diagram). The results demonstrated that Dof proteins from all three lineages clustered within shared clades, without forming distinct lineage-specific branches. This pattern indicates a high degree of sequence conservation of *Dof* genes between cultivated and wild-type flax varieties. Dof protein sequences from four dicotyledonous species (flax, *Arabidopsis* sp., soybean, and cassava) and two monocotyledonous species (rice and sorghum) were used to construct a phylogenetic tree to comparatively assess the evolutionary relationships among Dof proteins across diverse plant lineages (Figure 4). The resulting phylogenetic tree resolved 12 well-supported groups, with Group I containing the highest number of Dof proteins (*n* = 59) and Group IX comprising the fewest (*n* = 6). Among these 12 groups, Group X contained the highest number of LuDof members (*n* = 10), whereas Group XII contained no LuDof proteins. Groups II, IV, and IX were composed exclusively of Dof proteins from dicotyledonous species, whereas the other groups included members from both dicotyledonous and monocotyledonous lineages. Additionally, the Dof proteins from flax exhibited closer evolutionary relationships with those of soybean and cassava.

### 2.5. LuDof Gene Promoter Analysis

**Figure 5:**
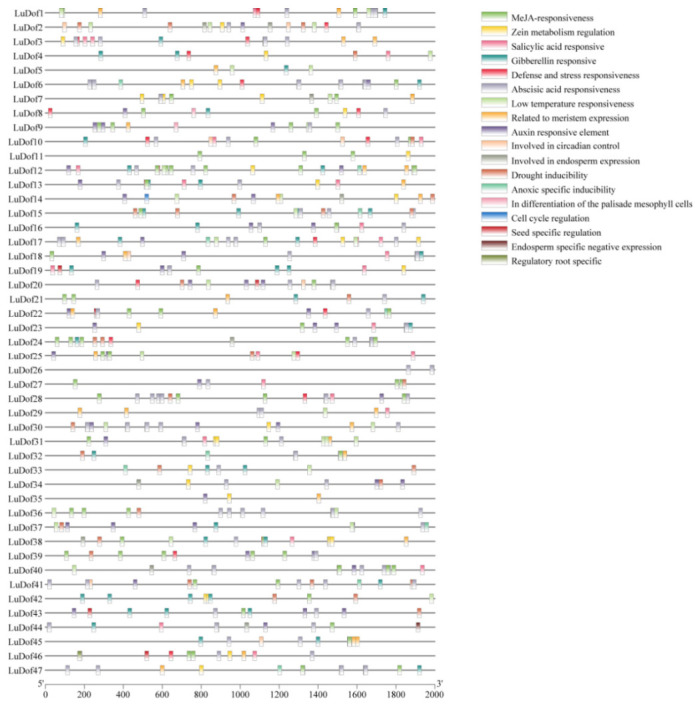
Distribution of *cis*-acting elements in the promoters of *LuDof* genes. Different colored boxes represent distinct *cis*-acting elements.

The regulatory potential and biological functions of *LuDof* genes were further elucidated through systematic characterization of *cis*-acting elements present in the promoter regions of all *LuDof* gene members. Promoter analysis revealed a diverse array of *cis*-acting elements associated with plant growth and development, phytohormone-mediated signaling, light responsiveness, and abiotic stress responses. Five major classes of hormone-responsive *cis*-acting elements were identified (Figure 5 and Table S7), corresponding to salicylic acid (SA), gibberellin (GA), auxin, methyl jasmonate (MeJA), and abscisic acid (ABA) responses. Among these, MeJA-responsive elements were the most prevalent, with 160 occurrences detected across 36 *LuDof* genes. In contrast, ABA-responsive elements exhibited the highest gene-level prevalence, being present in 40 genes and accounting for 146 instances in total. In contrast, auxin-, GA-, and SA-responsive elements were detected in 51, 47, and 29 instances, respectively, across the promoter regions of all *LuDof* genes. Notably, nine *LuDof* genes, namely, *LuDof12*, *LuDof13*, *LuDof16*, *LuDof17*, *LuDof18*, *LuDof19*, *LuDof23*, *LuDof38*, and *LuDof44*, harbored all five categories of hormone-responsive *cis*-acting elements. Quantitative assessment further revealed that *LuDof12* exhibited the highest abundance and diversity of *cis*-regulatory elements, with a total of 27 elements representing 10 distinct classes. Furthermore, approximately half of the *LuDof* genes harbored *cis*-elements associated with low-temperature responsiveness and drought inducibility. Additionally, *cis*-acting elements associated with the regulation of zein metabolism and meristem-specific expression were widely distributed across the *LuDof* gene family. In contrast, cell cycle-related *cis*-elements were exclusively identified in the promoter region of *LuDof14*, whereas endosperm-specific negative regulatory elements were uniquely present in *LuDof44*, and root-specific *cis*-elements were solely detected in the *LuDof4* promoter.

### 2.6. LuDof Gene Expression Across Different Cultivars, Tissues, and Stress Conditions

**Figure 6:**
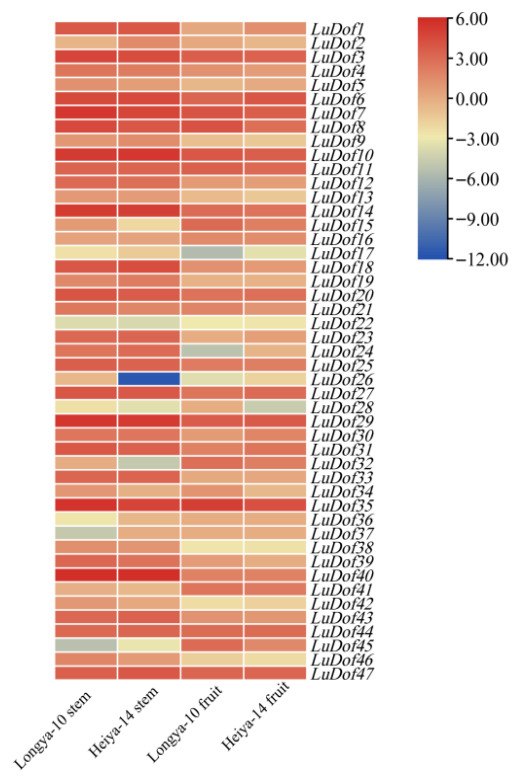
Expression analysis of *LuDof* genes across different flax varieties and tissues based on transcriptome data (data were log2-transformed). Unit is FPKM, and each plot represents three biological replicate samples.

This study utilized previously published transcriptome data to analyze plant tissues, systematically identifying the expression profiles of *LuDof* genes in different flax tissues and varieties, resulting in the successful retrieval of expression data for 47 *LuDof* genes. As depicted in Figure 6, several *LuDof* genes exhibited distinct tissue-specific expression patterns, with consistently higher expression in fruits than in stems across both flax varieties. Specifically, *Ludof15*, *Ludof16*, *Ludof32*, *Ludof41*, and *Ludof45* exhibited significantly higher expression in the high-oil Longya-10 variety compared to that in the high-fiber Heiya-14 cultivar. Conversely, the expression levels of 34 genes, including *LuDof1*, *LuDof3*–*LuDof10*, *LuDof12*–*LuDof14*, *LuDof17*–*LuDof21*, *LuDof23*–*LuDof25*, *LuDof27*, *LuDof29*–*LuDof31*, *LuDof33*, *LuDof35*, *LuDof38*–*LuDof40*, *LuDof42*–*LuDof44*, and *LuDof46*–*LuDof47*, were consistently higher in stems than in fruits in both flax cultivars. Of these, *Ludof1*, *Ludof6*, *Ludof9*, *Ludof10*, *Ludof17*–*Ludof19*, *Ludof23*, *Ludof24*, *Ludof30*, *Ludof33*, *Ludof43*, *Ludof44*, and *Ludof47* exhibited significantly higher expression in the high-fiber Heiya-14 cultivar than in the high-oil Longya-10 cultivar.

**Figure 7:**
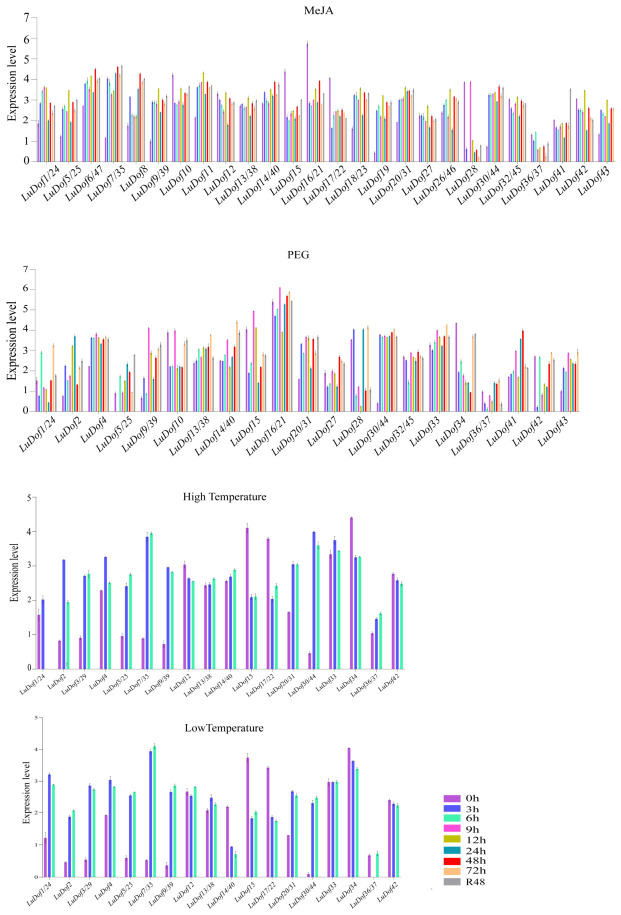
The expression levels of the *LuDof* gene in the leaves under different stress treatments were detected by qRT-PCR. The *x*-axis represents the time points (0, 3, 6, 9, 12, 24, 48, 72 h) after the different stress treatments and 48 h after recovery in 1/2 MS liquid medium without coercion supplementation, while the *y*-axis indicates the gene expression levels. Relative expression levels were calculated using the 2^-∆∆Ct^ method, followed by logarithmic transformation (log10). All results were derived from three biological replicates, with error bars representing ± SD (*n* = 3).

In this study, the expression profile of the *LuDof* gene under stress conditions was detected using quantitative polymerase chain reaction (qPCR) technology. The functional roles of *LuDof* genes were further elucidated by analyzing the expression profiles of genes harboring drought-, temperature- and MeJA-responsive *cis*-elements within their promoter regions under various abiotic and hormonal stress conditions, including polyethylene glycol (PEG)-induced drought stress, cold stress (4 °C), heat stress (42 °C), and treatment with exogenous MeJA. Quantitative gene expression analyses revealed distinct, stress-specific transcriptional responses among *LuDof* genes (Figure 7). Treatment with exogenous MeJA significantly induced nearly all the *LuDof* genes at least once during the time course, with the exception of *LuDof10*, *LuDof12*, *LuDof15*, *LuDof16/21*, and *LuDof17/22*. Notably, *LuDof1/24*, *LuDof5/25*, *LuDof6/47*, *LuDof7/35*, *LuDof8*, *LuDof9/39*, *LuDof11*, *LuDof14/40*, *LuDof18/23*, *LuDof19*, *LuDof20/31*, *LuDof30/44*, and *LuDof43* were significantly induced at all sampling time points, with peak expression for most *LuDof* genes occurring at 12 h or 48 h. With the exception of *LuDof34*, all the *LuDof* genes were significantly induced under PEG-induced drought stress at least once during the time course. Notably, *LuDof16/21* consistently exhibited the highest expression levels across all time points, reaching a peak at 9 h and surpassing the expression levels of all other *LuDof* genes analyzed. Furthermore, *LuDof2*, *LuDof4*, *LuDof9/39*, *LuDof13/38*, *LuDof20/31*, *LuDof30/44*, and *LuDof43* were consistently upregulated at all sampling points following treatment. In contrast, the remaining *LuDof* genes exhibited dynamic, time-dependent expression profiles, with certain genes showing transient induction, whereas the others were downregulated. Notably, the number of downregulated genes exceeded that of the upregulated genes relative to that at 0 h. The expression levels of all the *LuDof* genes were significantly upregulated under heat stress at all time points compared to those at 0 h, with the exception of *LuDof1/24*, *LuDof12*, *LuDof15*, *LuDof17/22*, *LuDof34*, and *LuDof42*. Among the heat-responsive *LuDof* genes, *LuDof30/44* exhibited peak expression at 3 h, whereas *LuDof7/35* reached peak expression at 6 h. *LuDof1/24*, *LuDof2*, *LuDof3/29*, *LuDof4*, *LuDof5/25*, *LuDof7/35*, *LuDof9/39*, *LuDof13/38*, *LuDof20/31*, and *LuDof30/44* were consistently upregulated under cold stress across all sampling time points. In contrast, *LuDof14/40*, *LuDof15*, *LuDof17/22*, *LuDof34*, and *LuDof42* exhibited sustained downregulation throughout the treatment period under cold stress. Notably, the findings further revealed that *LuDof7/LuDof35* exhibited the highest expression levels among all cold-responsive *LuDof* genes.

At the same time, we conducted a significance difference analysis on the qPCR results, and the results are presented in Supplementary Table S6. After JA treatment, the majority of samples showed extremely significant upregulation at all time points (*p* < 0.001). Only a few samples (such as *Dof32/45*) showed downregulation or no significant change at some time points. After PEG treatment, almost all samples showed highly significant downregulation at all time points (*p* < 0.001). The ΔCq values increased significantly, indicating that the expression levels were strongly suppressed. After high-temperature treatment, the expression trends were inconsistent. Approximately 70% of the samples showed significant downregulation, approximately 30% of the samples (such as *Dof7/35* and *Dof4*) showed significant upregulation, and a few samples showed no significant difference. After low-temperature treatment, almost all samples showed highly significant downregulation at all time points (*p* < 0.001). The ΔCq values increased significantly, indicating that the expression levels were strongly suppressed.

## 3. Discussion

The *Dof* gene family represents a plant-specific TF family that plays crucial roles in the regulation of plant growth, development, and abiotic stress adaptation. Although *Dof* genes have been extensively characterized across diverse plant species, a comprehensive genome-wide identification and systematic characterization of the *Dof* family in flax remains lacking. Consequently, the regulatory functions of *Dof* genes in the development and stress responses of flax remain poorly understood. To address this knowledge gap, we performed a comprehensive genome-wide identification of *Dof* genes in flax, followed by their phylogenetic classification and structural characterization—including analyses of exon–intron organization and conserved domain architecture—as well as spatiotemporal expression profiling. This study establishes a foundational resource for future mechanistic investigations of *LuDof* genes in the growth, development and stress adaptation of flax.

The present study identified 47 and 48 *Dof* genes in the cultivated flax varieties Longya-10 and Heiya-14, respectively, whereas their wild-type progenitor, pale flax, harbored 45 *Dof* genes. The near-identical copy numbers across the three flax genomes suggest that the *Dof* gene family has undergone minimal expansion during the domestication of flax, reflecting strong evolutionary conservation. Subcellular localization prediction revealed that all flax Dof proteins in flax exclusively localize to the nucleus, consistent with the well-established nuclear localization of Dof TFs across diverse plant species. With the exception of *LuDof45*, all the *LuDof* genes contained zero to two introns, a structural feature that is highly conserved across diverse plant lineages, including *Cerasus humilis* [29], rice [30], maize [31], and pepper [32], thus further highlighting the evolutionary stability of the *Dof* gene family. Intron retention and alternative splicing are well-documented mechanisms contributing to the expansion of transcriptomic and proteomic diversity in plants. Therefore, the presence of introns within *LuDof* genes may enhance their functional versatility in regulating developmental processes and mediating stress responses in flax [33]. Conserved motif analysis revealed that Motif 1, a hallmark feature present in nearly all LuDof proteins, corresponds to the canonical DNA-binding domain of Dof TFs. In contrast, lineage- or paralog-specific motifs, including Motifs 4, 18, and 48, were detected only in specific subsets of LuDof members, suggesting their potential involvement in functional diversification and subfunctionalization within the gene family.

In this study, three phylogenetic trees were constructed using the Dof proteins from flax and representative monocotyledonous and dicotyledonous plant species. The findings revealed consistent evolutionary relationships among the LuDof proteins, thereby validating the robustness and reliability of the phylogenetic inference. Notably, Group XII lacked LuDof members, suggesting a lineage-specific loss of *Dof* genes during the evolutionary trajectory of flax. Groups II, IV, and IX consisted exclusively of Dof proteins from eudicot species, whereas the remaining groups included Dof homologs from monocot and eudicot lineages. This distribution pattern suggests a high degree of functional conservation of Dof proteins across major angiosperm lineages, with potential specialization within eudicot-specific regulatory networks. Consistent with this observation, flax Dof proteins exhibited the closest phylogenetic affinity to those of soybean and cassava, supporting their conserved functions in the development and stress response pathways of eudicot species. Additionally, LuDof proteins clustered within the same phylogenetic group exhibited highly conserved gene structures and domain architectures, a feature that has also been reported in Dof families from other plant species [34]. Gene duplication represents a major driver of gene family expansion. Gene duplication analysis revealed that 42 *LuDof* genes in flax underwent duplication through SD events during evolution, suggesting that the expansion of the *LuDof* family was primarily driven by SD. Furthermore, all identified paralogous *LuDof* gene pairs exhibited Ka/Ks ratios < 1, providing strong evidence that the *LuDof* gene family has been subjected to pervasive purifying selection throughout its evolutionary history. Cross-species collinearity analysis further revealed that *Dof* genes are highly evolutionarily conserved across angiosperms. Specifically, flax *Dof* genes formed 120 syntenic orthologous gene pairs with soybean, a eudicot species, but only 10 such pairs with maize, a monocot species. This pronounced asymmetry in synteny density reflects the greater phylogenetic divergence between monocots and eudicots, and is consistent with extensive gene loss, duplication, and genomic rearrangements that have occurred in both lineages following their evolutionary divergence [35].

Previous studies have demonstrated that the promoter regions of *Dof* genes harbor a wide array of *cis*-acting regulatory elements associated with phytohormone and abiotic stress responses, and that *Dof* genes exhibit conserved responsiveness to diverse abiotic stresses across plant species. For instance, the *GhDofA5.7*, *GhDofA7.4*, and *GhDofD11.3* genes in cotton have been shown to be significantly upregulated under low-temperature stress [36]. Similarly, *BraDof023*, *BraDof045*, and *BraDof074* in *Brassica rapa* show marked upregulation in response to drought and salinity stress, whereas *BraDof003*, *BraDof023*, *BraDof045*, and *BraDof074* are induced by cold stress, and *BraDof072* is specifically downregulated under the same conditions [37]. It has been additionally reported that the majority of *PeDof* genes in passion fruit are heat inducible [38]. *Cis*-acting within gene promoters play a pivotal role in regulating gene expression in plants. The present study revealed that the promoter regions of *LuDof* genes harbored a wide array of hormone-responsive elements, including 44, 26, 79, and 137 elements associated with responses to GA, SA, MeJA, and ABA, respectively, as well as a diverse array of stress-responsive elements. Notably, each *LuDof* gene harbored one hormone-responsive and one stress-responsive element. These findings align closely with previous reports on the composition of *cis*-acting elements in the promoter regions of orthologous *Dof* genes in wheat [39], lotus [40] and *Populus simonii* [41]. In pea plants, researchers cloned and identified a novel Dof transcription factor named PsDof1 [42]. It was found that the PsDof1 protein could bind with high affinity to its own promoter fragment containing the AAAG sequence. This provides a rigorous demonstration of the transcriptional autoregulation mechanism of a Dof factor. Collectively, these results support the hypothesis that the expression of *LuDof* genes is modulated by upstream signaling molecules, thereby enabling LuDof TFs to function as regulatory nodes that coordinate both transcriptional autoregulation and the expression of downstream target genes. Additionally, the presence of diverse *cis*-acting elements likely facilitates the integration of various stress signals, thereby enhancing plant resilience to complex and concurrent environmental stresses.

The functional roles of *LuDof* genes in abiotic stress responses were further elucidated through comprehensive expression profiling under drought and cold stresses, with particular emphasis on genes harboring the corresponding *cis*-acting elements, namely, drought- or cold-responsive elements. Potential cross-temperature responsiveness was assessed by analyzing the expression of *LuDof* genes harboring cold-responsive *cis*-regulatory elements under heat stress. Given the predominance of MeJA-responsive elements among the predicted *cis*-acting elements, we further evaluated the expression dynamics of *LuDof* genes containing these elements in response to treatment with MeJA. The results demonstrated that the majority of *LuDof* genes analyzed herein were significantly upregulated in response to treatment with MeJA, suggesting their sensitivity to MeJA signaling. This expression pattern is consistent with observations in *Arabidopsis* sp., where the abundance of the *AtDof1.1* transcript was found to increase two- to three-fold following the application of MeJA [43,44]. Previous studies have demonstrated the conserved role of Dof genes in abiotic stress tolerance. For instance, Zhao et al. demonstrated that *AcDOF22* in kiwifruit contributes to drought tolerance [24], and another study similarly reported that CDF3, a key regulator of abiotic stress responses in plants, confers enhanced drought resistance in *Arabidopsis* sp. [45]. The present study demonstrated that PEG-induced drought stress significantly upregulated multiple *LuDof* genes, with *LuDof16/21* consistently exhibiting the highest expression is response to drought stress, suggesting that these genes likely function as pivotal regulators in the drought response network of flax. These findings highlight the evolutionarily conserved role of *Dof* genes in conferring drought resistance across diverse plant species [34]. Dof proteins have also been implicated in heat and cold stress responses. Previous studies have demonstrated that the protein encoded by *JrDOF3* in walnut physically interacts with the *JrGRAS2* gene product to enhance its transcriptional activity under heat stress [46]. whereas the *VaDof17d* gene in grape mediates cold stress resistance and represents a promising candidate gene for the molecular breeding of cold-resistant cultivars [47]. Consistent with these findings, the present study demonstrated that all analyzed *LuDof* genes exhibited significant, stress-specific transcriptional responses to both heat and cold stress. Notably, certain *LuDof* genes displayed qualitatively similar induction kinetics under both heat and cold stress conditions. Collectively, these results establish *LuDof* genes as integral regulators of thermotolerance and cold acclimation in flax. Notably, the *LuDof9/39*, *LuDof20/31*, and *LuDof30/44* gene pairs were significantly upregulated in a coordinated manner across all four abiotic stress conditions. This broad-spectrum, multi-stress responsiveness underscores their function as key regulators of integrated abiotic stress tolerance in flax and highlights their potential as high-priority candidates for molecular breeding strategies aimed at enhancing stress resilience in flax. Consistent with this notion, *Dof* genes exhibit evolutionarily conserved regulatory functions across diverse plant species under multiple stress conditions. For instance, the overexpression of *AtCDF3* in *Arabidopsis* sp. has been shown to confer enhanced tolerance to drought, cold, and osmotic stress [12,45]. Similarly, Wei et al. demonstrated that *GmDof41* overexpression in soybeans significantly improves tolerance to both drought and salinity stress [48]. Furthermore, tissue-specific expression profiling in the present study revealed that five *LuDof* genes are potentially involved in capsule development or fatty acid biosynthesis, whereas 14 *LuDof* genes exhibit preferential expression in stems, suggesting their plausible role in stem development. Based on expression analyses, these findings provide compelling evidence that *LuDof* genes serve as critical and multifaceted regulators of flax development and mediate adaptive responses to diverse abiotic stresses. Nevertheless, functional validation using genetic approaches, including CRISPR/Cas9-mediated knockout, overexpression, or promoter–reporter assays, remains essential to definitively establish the causal roles of individual *LuDof* genes in these processes.

## 4. Materials and Methods

### 4.1. Flax Cultivation and Induction of Stress

The Longya-10 variety of flax, developed in our laboratory, was used for the experiments in this study. To analyze the expression levels of *LuDof* genes, Longya-10 seeds were germinated in Petri dishes with water and cultivated in a climate-controlled chamber at a temperature of 25 °C under a 16 h/8 h light/dark photoperiod. After 14 days of growth, the uniformly developed seedlings were transferred to half-strength Murashige and Skoog (1/2 MS) liquid medium and cultured for an additional 4 days. The seedlings were subsequently subjected to salinity, drought, and hormone treatments by transferring to fresh medium supplemented with 200 mM NaCl, 20% (w/v) PEG, or 10 µM MeJA, respectively. The seedlings were additionally subjected to heat and cold stress at 42 °C and 4 °C, respectively. All plants used in the treatments were derived from a single sowing, and each treatment was conducted with three biological replicates. Leaf samples were harvested at 0, 3, and 6 h following cold and heat stress treatments; at 0, 3, 6, 9, 12, 24, 48, and 72 h following drought, salt, and hormone treatments; and after 48 h of additional culture in untreated medium. All the harvested samples were immediately frozen in liquid nitrogen and subsequently stored at −80 °C prior to RNA extraction.

### 4.2. Identification of the Dof Gene Family in Flax

Pale flax has a long cultivation history. It is generally believed to have originated in the Near East or along the Mediterranean coast; however, conclusive evidence is still lacking. The seeds of Pale flax used in this study have been preserved in our laboratory for many years. The variety ‘Heiya-14’ originates from Heilongjiang, China. The genomic sequences of the high-oil Longya-10 cultivar, the high-fiber Heiya-14 variety, and pale flax (*Linum bienne*) were assembled in our laboratory and deposited in NCBI under accession numbers QMEI00000000.2, QMEH00000000, and QMEG00000000, respectively. The corresponding genome annotation files have been made publicly available on Figshare (https://figshare.com/). The genome sequences for *Arabidopsis* sp., soybean (*Glycine max*), cassava (*Manihot esculenta*), rice, and Sorghum (*Sorghum bicolor*) were retrieved from Phytozome (v13, http://www.phytozome.net/). The Dof gene identifiers for *Arabidopsis* sp. [9], soybean [49], cassava [10], rice [30], and sorghum [50] were retrieved from previous studies, and the corresponding Dof protein sequences for each species were extracted from their respective genomic sequence data (Table S7). These Dof protein sequences were used as queries to identify the *Dof* gene families in Longya-10, Heiya-14, and pale flax through homology searches with BLASTp (https://blast.ncbi.nlm.nih.gov/Blast.cgi (accessed on 18 December 2025) (E-value = 1e-5)) using default parameters. The presence and integrity of conserved domains within the identified Dof proteins were subsequently verified using the NCBI Conserved Domain Database (CDD; https://www.ncbi.nlm.nih.gov/cdd/) and InterPro (https://www.ebi.ac.uk/interpro/ (accessed on 18 December 2025)). A non-redundant set of *LuDof* genes was finally generated by selecting a single representative gene model for each locus (Table S3).

### 4.3. Sequence Analyses

The MW, pI, and GRAVY of the proteins encoded by the *LuDof* genes were predicted using the online ExPASy ProtParam web server [51] (http://web.expasy.org/protparam/ (accessed on 19 December 2025)). The subcellular localization of the identified Dof proteins was predicted using the online Plant-mPloc web server [52] (http://www.csbio.sjtu.edu.cn/bioinf/plant-multi/ (accessed on 20 December 2025)). Conserved domain analysis was performed using the MEME Suite (http://alternate.meme-suite.org/tools/meme (accessed on 21 December 2025)) and the CD-search tool of NCBI, and the results were visualized using TBtools (v.2.146) [53]. *LuDof* gene structures were analyzed and visualized using Gene Structure Display Server (GSDS; http://gsds.cbi.pku.edu.cn/), whereas the *cis*-acting elements within the promoter regions of *LuDof* genes were predicted using the PlantCARE database (http://bioinformatics.psb.ugent.be/webtools/plantcare/html/ (accessed on 21 December 2025)).

### 4.4. Chromosomal Distribution, Gene Duplication Events, and Collinearity Analysis

The chromosomal positions of LuDof genes were extracted from the annotation file of the Longya-10 genome using TBtools, and gene identifiers were assigned based on their chromosomal distribution. The chromosomal positions of *LuDof* genes were subsequently visualized using the online MapGene2Chromosome web server (http://mg2c.iask.in/mg2c_v2.0/ (accessed on 23 December 2025)). Gene duplication events within the LuDof family, as well as the collinearity between *LuDof* genes and *Dof* genes from *Arabidopsis* sp., soybean, cassava, sorghum, and rice, were analyzed and visualized using TBtools. The Ka and Ks substitution rates per site for the duplicated gene pairs were calculated using DnaSP6 (Version 6) [54] software, and the selection pressures acting on these genes were inferred from their Ka/Ks ratios. The divergence time (Mya) between *LuDof* gene pairs was estimated using the formula: Divergence time (Mya) = (Ks/(2 × 6.1 × 10^−9^) ×) 10^−6^ [55].

### 4.5. Phylogenetic Analyses

The Dof protein sequences from six species, including flax (Longya-10), *Arabidopsis* sp., soybean, cassava, rice, and sorghum, were subjected to multiple sequence alignment using MEGA v6.0 [56], and a phylogenetic tree was constructed using the neighbor-joining (NJ) method with 1000 bootstrap replicates to assess branch support. An additional phylogenetic tree was constructed using Dof protein sequences from Longya-10, Heiya-14, and pale flax, applying the same NJ method with 1000 bootstrap replicates.

### 4.6. LuDof Gene Expression Analysis Across Different Cultivars, Tissues, and Stress Conditions

The expression patterns of *LuDof* genes were initially analyzed across two distinct genetic backgrounds (high-oil and high-fiber varieties) and two different tissue types using previously published transcriptome data (accession ID: PRJNA505721) [57]. The expression profiles were visualized as heat maps generated with TBtools. The expression patterns of *LuDof* genes in response to various hormone and stress treatments were systematically investigated through qRT-PCR analysis. RNA was extracted using the RNA extraction kit (BIOMGA) according to the manufacturer’s instructions. The concentration and quality of the extracted RNA were assessed using a NanoDrop-2000 spectrophotometer. Subsequently, the RNA was reverse transcribed into cDNA using the TaKaRa reverse transcription kit. Quantitative real-time PCR (qRT-PCR) was performed using the TB Green™ Premix Ex Taq™ II (TaKaRa) fluorescent quantitative reagent kit. The PCR reaction mixture (20 μL) consisted of 10 μL of 2× mix, 1 μL each of upstream and downstream primers (10 μmol·L^−1^), 2 μL of cDNA template, and 6 μL of RNase-free water. The qRT-PCR procedure was carried out as follows: 50 °C for 2 min, 95 °C for 10 min, followed by 40 cycles of 95 °C for 15 s, 60 °C for 15 s, and 72 °C for 15 s. The relative expression levels of *LuDof* genes were calculated using the 2^−ΔΔCt^ method, with three technical replicates per sample, and *GAPDH* was employed as the reference gene [58,59,60]. The expression data were log_10_-transformed, and samples collected at 0 h served as the control. The specific primers used for qRT-PCR are provided in Table S8. After the PCR results were exported, the data were organized and graphs were created using Prism 7 [61], Adobe Illustrator (Version 26.x) [62] software was used for image editing.

## 5. Conclusions

In this study, 47 *LuDof* genes were identified in the high-oil Longya-10 flax cultivar, and their physicochemical properties, conserved protein motifs, phylogenetic relationships, *cis*-acting promoter elements, and expression profiles were comprehensively characterized. Gene duplication analysis indicated that the expansion of the *LuDof* family was predominantly driven by SD events. Promoter analyses further revealed significant enrichment of stress- and hormone-responsive *cis*-elements in the promoter regions of *LuDof* genes. Subsequent qRT-PCR validation confirmed that the majority of *LuDof* genes were significantly induced under various stress conditions. Transcriptome-based expression profiling further revealed distinct tissue-specific expression patterns. Collectively, these integrated analyses provide strong evidence that *LuDof* genes are evolutionarily conserved, developmentally regulated, and stress-responsive transcriptional regulators that play central roles in the growth, development, and environmental adaptation of flax. Further research can enhance our understanding of plant stress responses and may provide information for future crop improvement strategies. These findings not only expand our understanding of the *Dof* gene family but also lay the foundation for a deeper understanding of their contribution to plant stress resistance. Overall, this work identifies a new *Dof* gene and reveals previously unresolved aspects of its evolutionary history, providing a new research approach for future analyses of other genes in this family.

## Figures and Tables

**Table 1:** Basic characteristics of LuDof family members.

Gene Name	Locus ID	Chromosome Location	Length (aa)	GRAVY	pI	MW (Da)
*LuDof1*	L.us.o.m.scaffold91.89	chr1:3778869-3779669(+)	266	−0.809	5.97	29,321.14
*LuDof2*	L.us.o.m.scaffold73.75	chr1:21766365-21767165(+)	266	−0.458	8.04	26,981.69
*LuDof3*	L.us.o.m.scaffold38.138	chr2:3907433-3909284(−)	330	−1.041	7.33	36,249.6
*LuDof4*	L.us.o.m.scaffold33.7	chr2:14078876-14079949(−)	171	−0.761	8.93	18,483.96
*LuDof5*	L.us.o.m.scaffold50.42	chr2:15660291-15662527(+)	336	−0.553	9.75	34,758.55
*LuDof6*	L.us.o.m.scaffold41.299	chr2:17397905-17398765(−)	286	−0.673	7.64	30,163.97
*LuDof7*	L.us.o.m.scaffold59.130	chr2:18264390-18267113(+)	468	−0.779	8.15	50,925.86
*LuDof8*	L.us.o.m.scaffold257.24	chr2:19876466-19878546(−)	401	−0.819	6.26	43,120.17
*LuDof9*	L.us.o.m.scaffold80.191	chr3:1675697-1677618(+)	368	−0.84	8.22	40,125.8
*LuDof10*	L.us.o.m.scaffold174.61	chr3:8329805-8331424(−)	283	−0.56	6.65	30,318.76
*LuDof11*	L.us.o.m.scaffold169.115	chr3:14283930-14284502(+)	190	−1.118	8.95	21,313.64
*LuDof12*	L.us.o.m.scaffold92.148	chr3:21634232-21636929(−)	358	−0.954	6.38	38,651.7
*LuDof13*	L.us.o.m.scaffold4.154	chr4:12375647-12376966(−)	397	−0.671	9.01	42,188.6
*LuDof14*	L.us.o.m.scaffold30.25	chr4:19607003-19607800(+)	303	−0.786	9.11	32,957.24
*LuDof15*	L.us.o.m.scaffold30.24	chr4:19642824-19643690(+)	263	−0.397	9.28	26,827.64
*LuDof16*	L.us.o.m.scaffold77.140	chr5:768899-771100(−)	414	−0.657	6.21	45,359.8
*LuDof17*	L.us.o.m.scaffold17.377	chr5:3809388-3810502(+)	272	−0.531	4.45	29,372.25
*LuDof18*	L.us.o.m.scaffold35.18	chr5:7476291-7477560(+)	305	−0.593	5.26	31,374.33
*LuDof19*	L.us.o.m.scaffold2.599	chr5:16682082-16683539(+)	332	−0.667	9.07	35,553.37
*LuDof20*	L.us.o.m.scaffold15.73	chr5:20314144-20319054(−)	446	−0.578	8.42	48,116.25
*LuDof21*	L.us.o.m.scaffold102.82	chr6:345304-347768(−)	413	−0.673	7.06	45,338.81
*LuDof22*	L.us.o.m.scaffold60.89	chr6:2918048-2918890(+)	280	−0.534	4.72	30,173.07
*LuDof23*	L.us.o.m.scaffold11.206	chr6:5209688-5210638(−)	316	−0.665	5.7	32,543.36
*LuDof24*	L.us.o.m.scaffold23.177	chr7:13704406-13705227(−)	273	−0.761	5.56	29,950.8
*LuDof25*	L.us.o.m.scaffold218.10	chr8:14755630-14758197(−)	345	−0.555	9.47	35,990.07
*LuDof26*	L.us.o.m.scaffold72.164	chr9:2927733-2930423(+)	335	−0.665	8.12	36,390.28
*LuDof27*	L.us.o.m.scaffold51.62	chr9:5168899-5170216(+)	273	−0.627	7.02	29,364.9
*LuDof28*	L.us.o.m.scaffold205.33	chr9:16802962-16803657(+)	231	−0.386	4.91	25,177.08
*LuDof29*	L.us.o.m.scaffold9.396	chr10:2434356-2435746(+)	324	−1.021	7.36	35,575.84
*LuDof30*	L.us.o.m.scaffold84.237	chr11:1698976-1700200(−)	257	−0.706	8.81	28,443.87
*LuDof31*	L.us.o.m.scaffold76.42	chr11:16692859-16694210(−)	313	−0.731	9.04	33,717.13
*LuDof32*	L.us.o.m.scaffold166.143	chr12:4848407-4849647(+)	347	−0.776	8.61	37,737.47
*LuDof33*	L.us.o.m.scaffold71.34	chr13:2002320-2003669(+)	292	−0.718	9.38	31,613.09
*LuDof34*	L.us.o.m.scaffold24.244	chr13:8980073-8981059(+)	328	−0.588	9.36	34,927.74
*LuDof35*	L.us.o.m.scaffold147.27	chr13:10528241-10531143(−)	470	−0.805	6.78	51,215.01
*LuDof36*	L.us.o.m.scaffold161.127	chr14:14909327-14910193(+)	288	−0.709	6.35	31,365.54
*LuDof37*	L.us.o.m.scaffold161.126	chr14:14914113-14915000(+)	295	−0.725	6.03	31,930.01
*LuDof38*	L.us.o.m.scaffold52.310	chr14:16002742-16004877(+)	378	−0.608	8.96	40,226.82
*LuDof39*	L.us.o.m.scaffold97.92	chr15:1866701-1868743(+)	354	−1.008	8.26	38,483.76
*LuDof40*	L.us.o.m.scaffold107.14	chr15:7517738-7519115(−)	282	−0.763	8.92	30,406.28
*LuDof41*	L.us.o.m.scaffold107.13	chr15:7564148-7565772(−)	267	−0.375	9.43	27,049.78
*LuDof42*	L.us.o.m.scaffold107.114	chr15:7657546-7658064(+)	172	−0.78	9.07	18,555.09
*LuDof43*	L.us.o.m.scaffold156.91	scaffold156:91517-93798(+)	349	−0.966	9.49	38,230.04
*LuDof44*	L.us.o.m.scaffold253.1	scaffold253_ERROPOS72288:64510-65740(+)	252	−0.707	9.00	27,751.09
*LuDof45*	L.us.o.m.scaffold281.14	scaffold281_ERROPOS116577:63558-64607(−)	349	−0.714	8.13	37,519.13
*LuDof46*	L.us.o.m.scaffold378.21	scaffold378:68446-69574(−)	345	−0.557	7.32	37,094.27
*LuDof47*	L.us.o.m.scaffold395.19	scaffold395:66309-67178(+)	289	−0.692	7.62	30,716.63

Note: *LuDof43-47* were not mapped on any chromosome; the subcellular localization showed that they were all in the cell nucleus.

**Table 2:** Duplication events, selection pressure, and divergence time of *LuDof* genes.

Gene Pairs	Duplication Event	Ka	Ks	Ka/Ks	Selection Type	Divergence Time (Mya)
*LuDof1/LuDof6*	SD	0.322	0.969	0.332	Purifying selection	79.451
*LuDof1/LuDof24*	SD	0.265	1.192	0.222	Purifying selection	97.730
*LuDof3/LuDof29*	SD	0.074	0.453	0.162	Purifying selection	37.115
*LuDof3/LuDof31*	SD	0.142	0.776	0.183	Purifying selection	63.639
*LuDof3/LuDof20*	SD	0.183	0.453	0.404	Purifying selection	37.090
*LuDof4/LuDof42*	SD	0.177	0.476	0.372	Purifying selection	39.033
*LuDof5/LuDof13*	SD	0.118	0.647	0.182	Purifying selection	53.049
*LuDof5/LuDof19*	SD	0.229	0.625	0.366	Purifying selection	51.213
*LuDof5/LuDof25*	SD	0.202	0.863	0.234	Purifying selection	70.770
*LuDof5/LuDof38*	SD	0.230	1.384	0.166	Purifying selection	113.475
*LuDof6/LuDof24*	SD	0.106	2.152	0.049	Purifying selection	176.393
*LuDof6/LuDof47*	SD	0.336	0.771	0.436	Purifying selection	63.230
*LuDof7/LuDof35*	SD	0.289	1.045	0.277	Purifying selection	85.664
*LuDof8/LuDof34*	SD	0.176	1.594	0.110	Purifying selection	130.631
*LuDof9/LuDof39*	SD	0.344	1.591	0.216	Purifying selection	130.443
*LuDof10/LuDof27*	SD	0.203	1.197	0.169	Purifying selection	98.123
*LuDof10/LuDof29*	SD	0.233	1.319	0.176	Purifying selection	108.139
*LuDof13/LuDof19*	SD	0.210	0.476	0.441	Purifying selection	39.016
*LuDof13/LuDof25*	SD	0.197	0.598	0.329	Purifying selection	49.008
*LuDof13/LuDof38*	SD	0.205	3.325	0.062	Purifying selection	272.533
*LuDof14/LuDof40*	SD	0.161	0.447	0.360	Purifying selection	36.615
*LuDof15/LuDof41*	SD	0.242	2.607	0.093	Purifying selection	213.697
*LuDof16/LuDof21*	SD	0.177	0.280	0.633	Purifying selection	22.918
*LuDof17/LuDof22*	SD	0.186	1.756	0.106	Purifying selection	143.967
*LuDof17/LuDof28*	SD	0.231	0.947	0.244	Purifying selection	77.598
*LuDof18/LuDof23*	SD	0.118	0.655	0.180	Purifying selection	53.648
*LuDof19/LuDof43*	SD	0.125	0.788	0.159	Purifying selection	64.557
*LuDof20/LuDof29*	SD	0.187	0.293	0.638	Purifying selection	23.984
*LuDof20/LuDof31*	SD	0.203	0.471	0.432	Purifying selection	38.566
*LuDof22/LuDof28*	SD	0.145	0.513	0.283	Purifying selection	42.033
*LuDof24/LuDof47*	SD	0.306	0.847	0.361	Purifying selection	69.402
*LuDof25/LuDof38*	SD	0.187	2.007	0.093	Purifying selection	164.467
*LuDof26/LuDof46*	SD	0.304	1.220	0.249	Purifying selection	99.975
*LuDof29/LuDof31*	SD	0.178	0.431	0.413	Purifying selection	35.287
*LuDof30/LuDof44*	SD	0.072	2.051	0.035	Purifying selection	168.098
*LuDof32/LuDof45*	SD	0.191	1.678	0.114	Purifying selection	137.508
*LuDof33/LuDof43*	SD	0.081	0.733	0.110	Purifying selection	60.107
*LuDof38/LuDof43*	SD	0.124	1.305	0.095	Purifying selection	106.926
*LuDof36/LuDof37*	TD	0.083	0.544	0.153	Purifying selection	44.574

Note: SD represents segmental duplication, and TD represents tandem duplication.

## Data Availability

The original contributions presented in this study are included in the article/Supplementary Materials. Further inquiries can be directed to the corresponding authors.

## Supplementary Materials

The following supporting information can be downloaded at: https://www.mdpi.com/article/10.3390/ijms27094126/s1.
